# MicroRNA-17-92 cluster promotes the proliferation and the chemokine production of keratinocytes: implication for the pathogenesis of psoriasis

**DOI:** 10.1038/s41419-018-0621-y

**Published:** 2018-05-11

**Authors:** Weigang Zhang, Xiuli Yi, Yawen An, Sen Guo, Shuli Li, Pu Song, Yuqian Chang, Shaolong Zhang, Tianwen Gao, Gang Wang, Chunying Li

**Affiliations:** Department of Dermatology, Xijing hospital, Fourth Military Medical University, Xi’an, Shannxi China

## Abstract

Keratinocytes are the main epidermal cell type that constitutes the skin barrier against environmental damages, which emphasizes the balance between the growth and the death of keratinocytes in maintaining skin homeostasis. Aberrant proliferation of keratinocytes and the secretion of inflammatory factors from keratinocytes are related to the formation of chronic inflammatory skin diseases like psoriasis. MicroRNA-17-92 (miRNA-17-92 or miR-17-92) is a miRNA cluster that regulates cell growth and immunity, but the role of miR-17-92 cluster in keratinocytes and its relation to skin diseases have not been well investigated. In the present study, we initially found that miR-17-92 cluster promoted the proliferation and the cell-cycle progression of keratinocytes via suppressing cyclin-dependent kinase inhibitor 2B (CDKN2B). Furthermore, miR-17-92 cluster facilitated the secretion of C-X-C motif chemokine ligand 9 (CXCL9) and C-X-C motif chemokine ligand 10 (CXCL10) from keratinocytes by inhibiting suppressor of cytokine signaling 1 (SOCS1), which enhanced the chemotaxis for T lymphocytes formed by keratinocytes. In addition, we detected increased expression of miR-17-92 cluster in psoriatic lesions and the level of lesional miR-17-92 cluster was positively correlated with the disease severity in psoriasis patients. At last, miR-17-92 cluster was increased in keratinocytes by cytokines through the activation of signal transducers and activators of transcription 1 (STAT1) signaling pathway. Our findings demonstrate that cytokine-induced overexpression of miR-17-92 cluster can promote the proliferation and the immune function of keratinocytes, and thus may contribute to the development of inflammatory skin diseases like psoriasis, which implicates miR-17-92 cluster as a potential therapeutic target for psoriasis and other skin diseases with similar inflammatory pathogenesis.

## Introduction

MicroRNAs (miRNAs) are a class of noncoding RNAs that suppress gene expression by targeting messenger RNAs (mRNAs), leading to their translational repression or, less frequently, degradation^1^. It is known that miRNAs can influence the growth, the differentiation and the death of cells and are thus involved in the pathogenesis of many diseases^2^. In 2004, researchers reported the discovery of a novel gene called chromosome 13 open reading frame 25 (*C13orf25*) that can transcribe miRNA-17-92 (miR-17-92), a miRNA cluster composed of six miRNAs including mir-17, miR-18a, miR-19a, miR-19b, miR-20a, and miR-92a^3^. It is reported that the six mature miRNAs derived from miR-17-92 cluster have convergent target genes and cooperatively regulates the growth of cells like neurons and endothelia^4,5^. Moreover, miR-17-92 cluster regulates the differentiation of lymphocytes and the function of both adaptive and innate immune cells^6–9^. Given its master regulatory role in cell growth and immunity, miR-17-92 cluster contributes to the development of diverse diseases including cancers and autoimmune diseases^10–14^.

Keratinocytes are the predominant cell type in the epidermis, accounting for 90% of the cells at the outermost layer of the skin^15^. The primary function of keratinocytes is to form a physical skin barrier against environmental damages, which emphasizes the balance between the growth and the death of keratinocytes in maintaining skin homeostasis^16^. As a matter of fact, aberrant proliferation of keratinocytes is involved in the formation of chronic inflammatory skin diseases including psoriasis, atopic dermatitis, and lichen planus^17–19^. Moreover, keratinocytes can act as innate immune cells and release a variety of inflammatory factors, particularly chemokines, which further aggravates those inflammatory skin diseases^20–22^. Previous studies have reported a series of miRNAs that regulate the growth or the immune function of keratinocytes^23–25^. Despite the vital function of miR-17-92 cluster in regulating cell growth and immunity, the role of miR-17-92 cluster in keratinocytes and its relation to skin diseases have not been well investigated.

To elucidate the effect of miR-17-92 cluster on keratinocyte growth and function, the current study initially constructed keratinocytes that overexpressed miR-17-92 using normal human keratinocytes (NHKs). We found that miR-17-92 cluster not only promoted the proliferation of keratinocytes but also facilitated the secretion of chemokines from keratinocytes. Subsequent experiments revealed the target genes responsible for the regulation of miR-17-92 cluster on the proliferation and the immune function of keratinocytes, respectively. Moreover, we examined the lesional level of miR-17-92 cluster in psoriasis, a common inflammatory skin disease, and further investigated the mechanism underlying in the expression of cutaneous miR-17-92 cluster in inflammatory microenvironment.

## Results

### MiR-17-92 cluster promotes the proliferation and the cell-cycle progression of keratinocytes

**Fig. 1:**
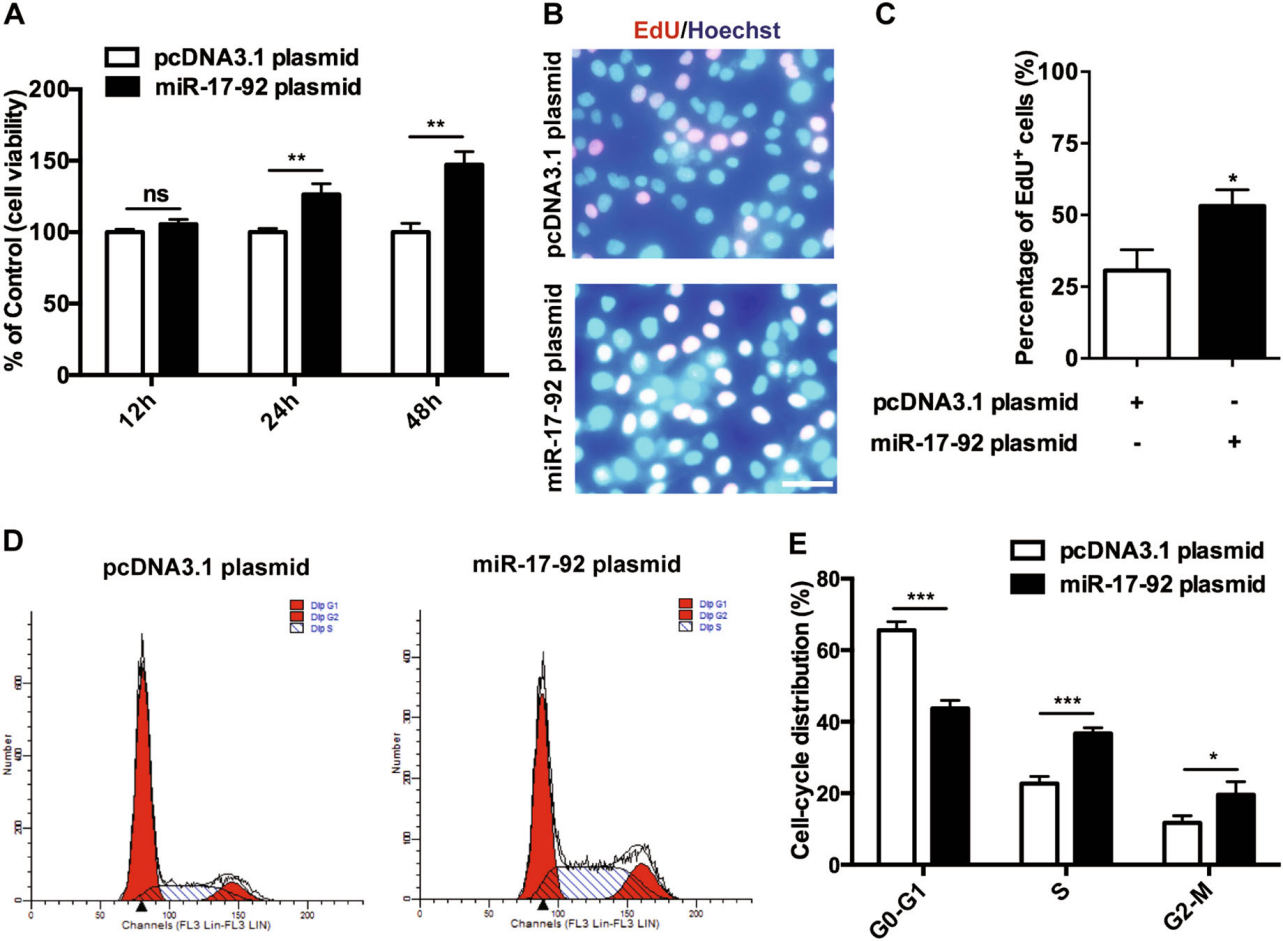
MiR-17-92 cluster promotes the proliferation and the cell-cycle progression of keratinocytes. **a** The cell viability of NHKs with different transfections as indicated for 12, 24, or 48 h was estimated using CCK8 assay. Values represent mean ± SD of three independent experiments. **P < 0.01; ns not significant. **b**, **c** Detection of EdU add-in cells (red) at 24 h after transfection. Nuclei were counterstained with Hoechst (blue). Scale bar = 50 μm. The bar graph represents mean ± SD of five wells. *P < 0.05. **d**, **e** Cell-cycle distributions of NHKs were analyzed 24 h after different transfections as indicated. The statistical chart represents mean ± SD of three individual experiments. *P < 0.05; ***P < 0.001

In order to investigate the effect of miR-17-92 cluster on the growth of keratinocytes, NHKs were transfected with miR-17-92 plasmid or the control pcDNA3.1 plasmid (transfection efficiency shown in Supplementary Figures S1A and B), respectively, and then examined by CCK8 assay. Although miR-17-92 overexpression failed to affect the growth of keratinocytes at 12 h after transfection, longer transfection time of both 24 and 48 h with miR-17-92 plasmid successfully promoted the viability of keratinocytes (Fig. 1a). To determine whether the increased cell viability was due to an increase of cell proliferation or a decrease of cell apoptosis, we assessed the proliferation and the apoptosis of the keratinocytes with different transfections using EdU assay and flow cytometry, respectively. As a result, the EdU incorporation rate (percentage of cells that undergo cell division) was elevated in the cells transfected with miR-17-92 plasmid compared with the control (Fig. 1b, c), while the apoptotic rate was not significantly changed (Supplementary Figures S2A and B). Moreover, miR-17-92 overexpression facilitated the cell-cycle progression of keratinocytes, with less cells arrested in the G0-G1 phase and more cells progressed to the S phase and G2-M phase (Fig. 1d, e). Collectively, our findings indicate that miR-17-92 cluster promotes the proliferation of keratinocytes, which probably results from the progressed cell cycle.

### CDKN2B suppression accounts for the enhanced proliferation and the progressed cell cycle of keratinocytes induced by miR-17-92 cluster

**Fig. 2:**
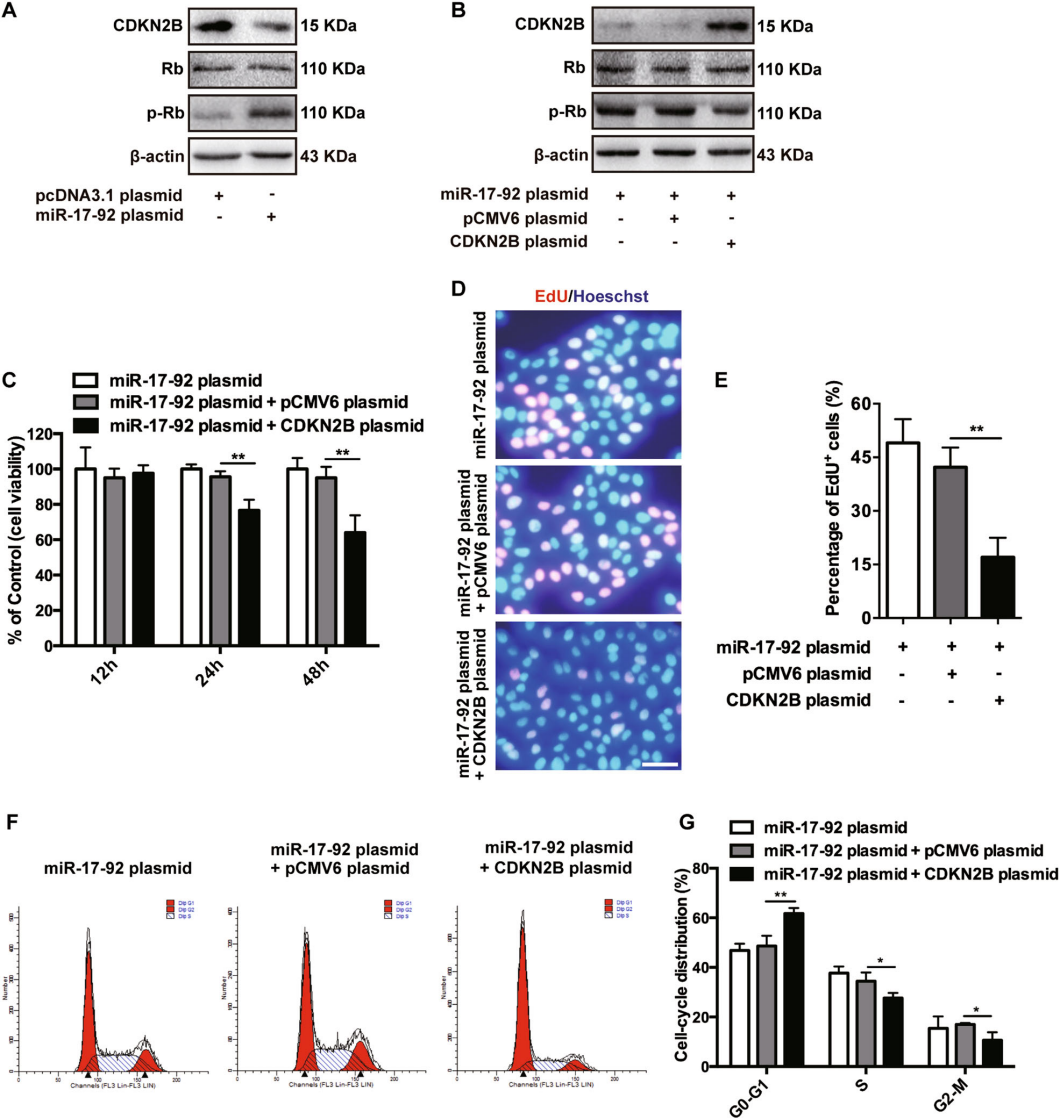
CDKN2B suppression accounts for the enhanced proliferation and the progressed cell cycle of keratinocytes induced by miR-17-92 cluster. **a**, **b** The levels of CDKN2B, Rb, and p-Rb in NHKs with different transfections as indicated were detected by western blotting. β-actin was detected as loading control. Data are representative of three independently performed experiments. **c** The cell viability of NHKs with different transfections as indicated for 12, 24, or 48 h was estimated using CCK8 assay. Values represent mean ± SD of three independent experiments. **P < 0.01. **d**, **e** Detection of EdU add-in cells (red) at 24 h after transfection. Nuclei were counterstained with Hoechst (blue). Scale bar = 50 μm. Bar graphs represent the mean ± SD of five wells. **P < 0.01. **f**, **g** Cell-cycle distributions of NHKs were analyzed 24 h after transfection. The statistical chart represents three individual experiments. Data are presented as means ± SD. *P < 0.05; **P < 0.01

A common bioinformatics tool Targetscan (http://www.targetscan.org) was used to find the potential target genes of miR-17-92 cluster responsible for its regulatory effect on keratinocytes. We found that four mature miRNAs derived from miR-17-92 cluster, including miR-17, miR-18a, miR-19a, and miR-19b, had been predicted to inhibit the expression of cyclin-dependent kinase inhibitor 2B (CDKN2B) via each complementary sequences (Supplementary Figures S3A). CDKN2B, also known as p15, is a tumor suppressor gene that prevents the activation of cyclin-dependent kinases (CDKs) and thus leads to cell-cycle arrest at G1 phase^26,27^, which made us speculate that miR-17-92 cluster could promote the proliferation of keratinocytes via inhibiting CDKN2B. To testify this, we first examined the expression of CDKN2B in keratinocytes transfected with miR-17-92 plasmid. It turned out that overexpressed miR-17-92 reduced the protein level of CDKN2B while increasing the phosphorylated protein level of retinoblastoma protein (Rb) (Fig. 2a), which was a sign of the activation of CDKs, because Rb can be phosphorylated by CDKs followed by the loss of its suppressing effect on cell-cycle progression^28^. We then performed luciferase reporter assays to verify whether miR-17-92 cluster repressed the expression of CDKN2B by targeting the predicted sequences in the 3′ untranslated regions (3′UTR) of CDKN2B mRNA. To this end, three parts of the 3′UTR (each with 300 nucleotides), with putative binding site for the mature miRNAs of miR-17-92 cluster involved in the middle, were inserted into the reporter plasmids and named CDKN2B WT1, CDKN2B WT2, and CDKN2B WT3, respectively, and corresponding plasmids named CDKN2B MUT1, CDKN2B MUT2, and CDKN2B MUT3 with the mutation of four or five nucleotides within the seed-matching sequences were also constructed (Supplementary Figures S3B-D). All the reporter plasmids were, respectively, co-transfected into NHKs with miR-17-92 plasmid or control plasmid, and luciferase activity was analyzed 48 h after transfection. The assays showed that miR-17-92 cluster reduced the luciferase activity in the cells transfected with CDKN2B WT1, CDKN2B WT2, or CDKN2B WT3, but failed to influence the reporter activity of CDKN2B MUT1, CDKN2B MUT2, and CDKN2B MUT3 (Supplementary Figures S3E). These results demonstrate that miR-17-92 cluster inhibits the expression of CDKN2B by the binding of the mature miRNAs to the predicted target sites in the 3′UTR of CDKN2B mRNA.

The inhibition of miR-17-92 cluster on CDKN2B expression encouraged us to investigate whether the decreased CDKN2B was required for the effect of miR-17-92 cluster on the proliferation and the cell cycle of keratinocytes. In order to investigate this, NHKs overexpressing miR-17-92 were co-transfected with 3′UTR-deleted CDKN2B plasmid or control pCMV6 plasmid. It turned out that the re-introduction of CDKN2B was accompanied with the downregulation of phosphorylated Rb as expected (Fig. 2b). Subsequent CCK8 and EdU assays showed that the restoring expression of CDKN2B inhibited the viability (Fig. 2c) and the proliferation (Fig. 2d, e) of keratinocytes pre-transfected with miR-17-92 plasmid. In addition, CDKN2B did invert the progressed cell-cycle distribution of keratinocytes induced by miR-17-92 overexpression (Fig. 2f, g). Taken together, these findings illustrate that miR-17-92 cluster promotes the proliferation and the cell-cycle progression of keratinocytes via inhibiting CDKN2B.

### MiR-17-92 cluster promotes the production of chemokines in keratinocytes primed by cytokines

**Fig. 3:**
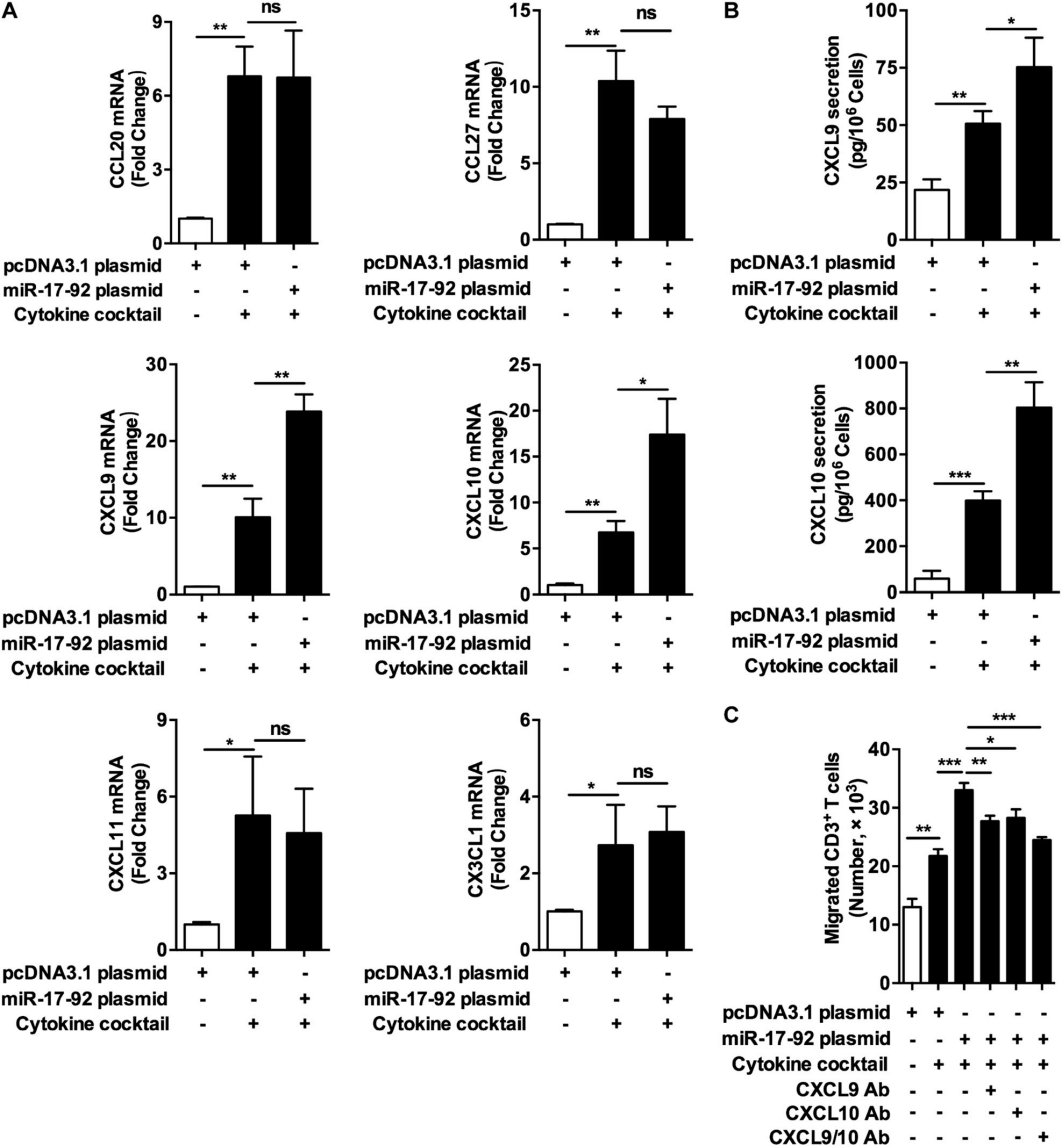
MiR-17-92 cluster promotes the production of chemokines in keratinocytes primed by cytokines. **a** The mRNA levels of CCL20, CCL27, CXCL9, CXCL10, CXCL11, and CX3CL1 in NHKs with different treatments and transfections as indicated were analyzed using qRT-PCR. Mean ± SD is shown. Data are representative of three independently performed experiments. *P < 0.05; **P < 0.01; ns not significant. **b** The culture mediums of NHKs with different treatments and transfections as indicated were analyzed by ELISA to determine the secretion levels of CXCL9 and CXCL10. Mean ± SD is shown. Data are representative of three independently performed experiments. *P < 0.05; **P < 0.01; ***P < 0.001. **c** The migrations of CD3^+^ T cells in response to the culture mediums from NHKs with different treatments and transfections as indicated were evaluated using transwell assay. Mean ± SD is shown. Data are representative of three individual experiments. *P < 0.05; **P < 0.01; ***P < 0.001

The production and the secretion of inflammatory factors from cytokines-stimulated keratinocytes, particularly the chemokines that induce the infiltration of immune cells, contribute to the development of a variety of inflammatory skin diseases, including psoriasis, vitiligo, and lichen planus^20,21,29^. Given that miR-17-92 cluster promotes the intracellular signaling pathways of multiple cytokines^30–32^, we considered that miR-17-92 cluster could promote the production of chemokines in keratinocytes treated by cytokines. To verify this hypothesis, we treated miR-17-92-overexpressing keratinocytes with a set of cytokine cocktail composed of tumor necrosis factor-alpha (TNF-α), interferon-gamma (IFN-γ), interleukin-17 (IL-17), and interleukin-22 (IL-22), and then examined the expressions of six chemokines using quantitative real-time polymerase chain reaction (qRT-PCR), including C-C motif chemokine ligand 20 (CCL20), C-C motif chemokine ligand 27 (CCL27), C-X-C motif chemokine ligand 9 (CXCL9), C-X-C motif chemokine ligand 10 (CXCL10), C-X-C motif chemokine ligand 11 (CXCL11), and C-X3-C motif chemokine ligand 1 (CX3CL1), all of which can mediate the infiltration of effector T cells to skin^33–36^. It turned out that the mRNA levels of the six chemokines all increased following cytokine stimulation as expected. Meanwhile, miR-17-92 cluster successfully promoted the expressions of CXCL9 and CXCL10 in keratinocytes stimulated by cytokines, though the remaining four chemokines showed no change (Fig. 3a). Consistently, using miR-17-92 siRNA to knock down the expression of miR-17-92 cluster (interference efficiency shown in Supplementary Figures S4A and B) attenuated the elevation of CXCL9 and CXCL10 mRNA levels in keratinocytes induced by cytokines (Supplementary Figures S4C). Further, enzyme-linked immunosorbent (ELISA) assay revealed that miR-17-92 overexpression accelerated cytokines-induced secretion of both CXCL9 and CXCL10 from keratinocytes (Fig. 3b), while miR-17-92 knockdown showed opposite effects (Supplementary Figures S4D). We went on to investigate whether miR-17-92 cluster could facilitate the T cell chemotaxis created by chemokines secreted from keratinocytes. Using flow cytometry, CD3^+^ T cells were sorted from the patients with psoriasis, which is a disease caused by cutaneous infiltration of T cells. Subsequent transwell assay showed that the culture supernatant of cytokines-treated keratinocytes induced the migration of T cells, and miR-17-92 overexpression further promoted this effect, which, however, were abolished by the addition of CXCL9 or CXCL10 neutralizing antibody into the transwell system (Fig. 3c), whereas miR-17-92 knockdown significantly weakened the chemotactic ability of the culture supernatant of cytokines-treated keratinocytes (Supplementary Figures S4E). Collectively, these findings support that miR-17-92 cluster promotes the production of chemokines in keratinocytes primed by cytokines and further enhances the T cell chemotaxis formed by keratinocytes.

### MiR-17-92 cluster promotes the production of chemokines in keratinocytes via suppressing SOCS1

**Fig. 4:**
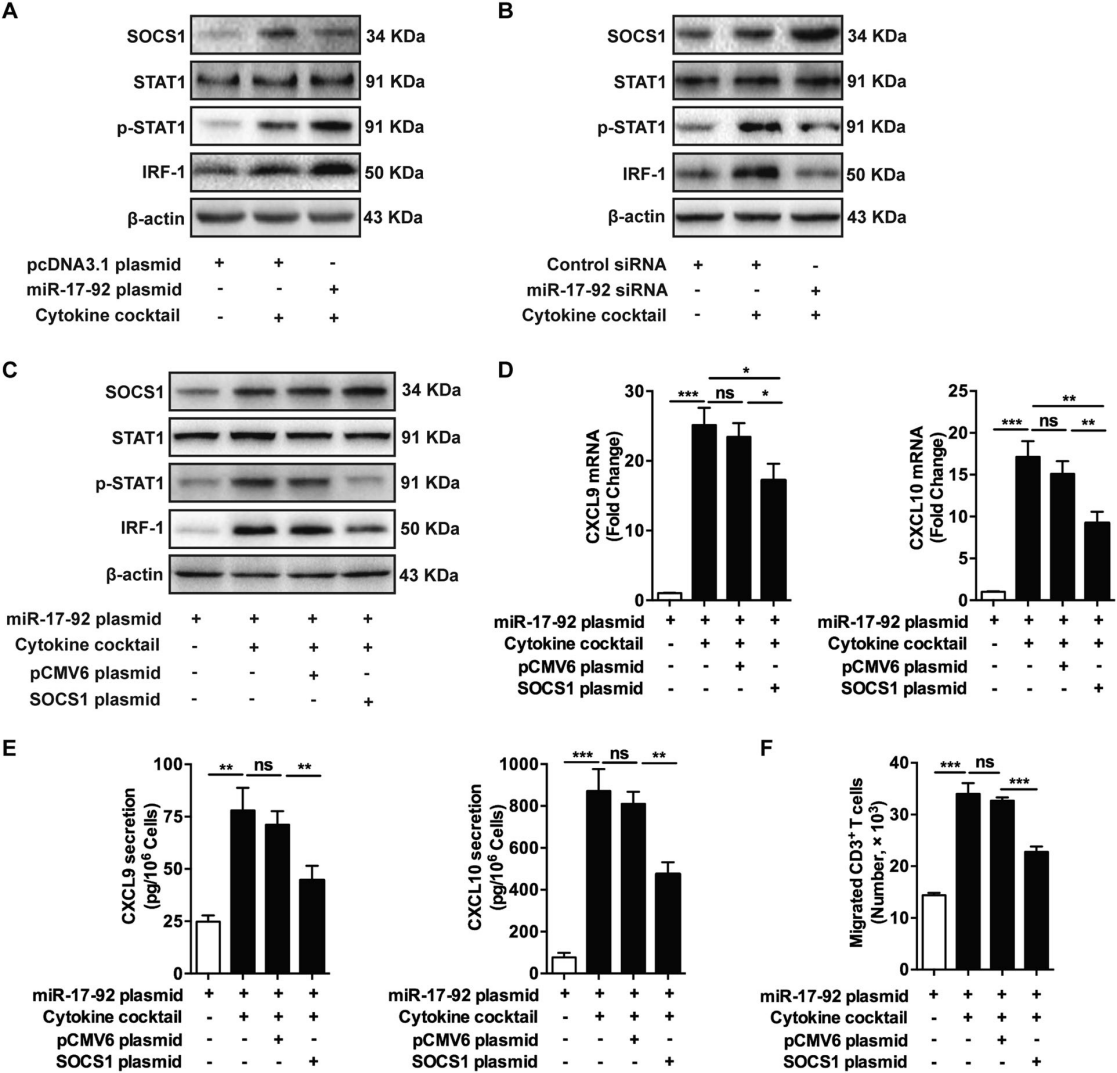
MiR-17-92 cluster promotes the production of chemokines in keratinocytes via suppressing SOCS1. **a**–**c** The levels of SOCS1, STAT1, p-STAT1, and IRF-1 in NHKs with different transfections and treatments as indicated were detected by western blotting. β-actin was detected as loading control. Data are representative of three independently performed experiments. **d** The mRNA levels of CXCL9 and CXCL10 in NHKs with different treatments and transfections as indicated were analyzed using qRT-PCR. Mean ± SD is shown. Data are representative of three individual experiments. *P < 0.05; **P < 0.01; ***P < 0.001; *ns* not significant. **e** The culture mediums of NHKs with different treatments and transfections as indicated were analyzed by ELISA to determine the secretion levels of CXCL9 and CXCL10. Mean ± SD is shown. Data are representative of three independently performed experiments. **P < 0.01; ***P < 0.001; *ns* not significant. **f** The migrations of CD3^+^ T cells in response to the culture mediums from NHKs with different treatments and transfections as indicated were evaluated using transwell assay. Mean ± SD is shown. Data are representative of three independently performed experiments. ***P < 0.001; *ns* not significant

We again used Targetscan to screen the potential target genes of miR-17-92 cluster that were responsible for the upregulation of CXCL9 and CXCL10 in keratinocytes primed by cytokines. Two of the six mature miRNAs derived from miR-17-92 cluster, miR-19a, and miR-19b had the potential to inhibit the expression of suppressor of cytokine signaling 1 (SOCS1) via each complementary sequences (Supplementary Figures S5A). SOCS1 is an immunosuppressing signaling regulator that inhibits the activation of signal transducers and activators of transcription 1 (STAT1) signaling pathway^37^. Given that CXCL9 and CXCL10 had been demonstrated to be upregulated following STAT1 activation^38^, we thought that miR-17-92 cluster probably facilitated the expression of the two chemokines in cytokines-stimulated keratinocytes via repressing SOCS1. To confirm this, we detected the protein level of SOCS1 in NHKs with miR-17-92 overexpression and the treatment of cytokine cocktail. The western blot analysis showed that the levels of phosphorylated STAT1, the downstream transcriptional factor interferon regulatory factor-1 (IRF-1)^39^, and the negative feedback regulator SOCS1 were all increased following cytokine stimulation, while miR-17-92 overexpression inhibited the expression of SOCS1 and further promoted the expressions of phosphorylated STAT1 and IRF-1 (Fig. 4a). In accordance with this, miR-17-92 knockdown further enhanced the upregulation of SOCS1 induced by cytokines and suppressed the protein levels of phosphorylated STAT1 and IRF-1 (Fig. 4b). We then constructed SOCS1 WT reporter plasmid with part of 3′UTR of SOCS1 mRNA (with 300 nucleotides) that had putative binding site for miR-19a/b in the middle, as well as corresponding SOCS1 MUT reporter plasmid with the mutation of five nucleotides within the seed-matching sequences (Supplementary Figures S5B). It turned out that miR-17-92 cluster reduced the luciferase activity in the cells transfected with SOCS1 WT, but failed to influence the reporter activity of SOCS1 MUT (Supplementary Figures S5C), which affirmed SOCS1 as a target gene for miR-17-92 cluster.

We further performed reverse experiment by using the co-transfections of miR-17-92 plasmid and SOCS1 plasmid in keratinocytes primed by cytokine cocktail. The re-introduction of SOCS1 was accompanied with the decrease of phosphorylated STAT1 and IRF1, though miR-17-92 was overexpressed in all the groups (Fig. 4c). Moreover, SOCS1 abrogated the enhancing effect of miR-17-92 cluster on the mRNA expressions (Fig. 4d) and the secretional levels (Fig. 4e) of CXCL9 and CXCL10 in keratinocytes primed by cytokine cocktail. Consistently, our transwell assay showed that under the stimulation of cytokine cocktail, the culture supernatant of keratinocytes transfected by both miR-17-92 and SOCS1 plasmids induced less migration of T cells compared with control (Fig. 4f). In summary, miR-17-92 cluster could promote the production of chemokines in keratinocytes primed by cytokines via suppressing SOCS1.

### The expression of miR-17-92 cluster is increased in psoriasis lesions

**Fig. 5:**
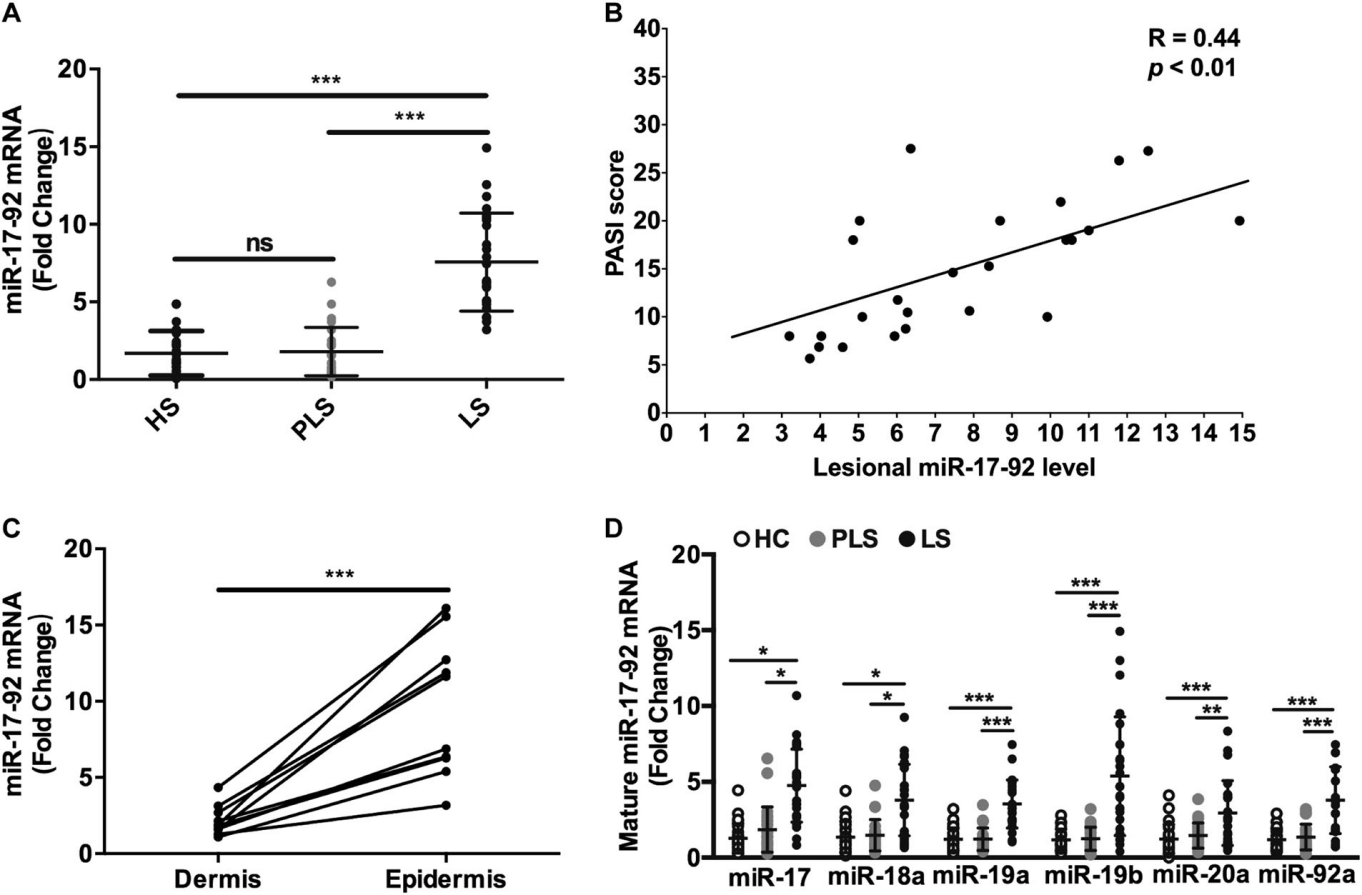
The expression of miR-17-92 cluster is increased in psoriasis lesions. **a** The level of miR-17-92 cluster in perilesional skin (PLS) tissues and lesional skin (LS) tissues from 25 psoriasis patients and healthy skin (HS) tissues from 25 healthy controls was analyzed using qRT-PCR. Mean ± SD is shown. ***P < 0.001; ns not significant. **b** The correlation between PASI score and lesional miR-17-92 level in 25 psoriasis patients was analyzed using Spearman’s rank correlation test. **c** The level of miR-17-92 cluster in perilesional epidermis tissues and lesional dermis tissues from 10 psoriasis patients was analyzed using qRT-PCR. Each oblique line links the data from the same patient. ***P < 0.001. **d** The levels of miR-17, miR-18a, miR-19a, miR-19b, miR-20a, and miR-92a in perilesional skin (PLS) tissues and lesional skin (LS) tissues from 25 psoriasis patients and healthy skin (HS) tissues from 25 healthy controls were analyzed using qRT-PCR. Mean ± SD is shown. *P < 0.05; **P < 0.01; ***P < 0.001

The regulatory effect of miR-17-92 cluster on the growth and inflammatory function of keratinocytes prompted us to evaluate whether this regulation could play a role in the development of common cutaneous diseases. We chose to focus on psoriasis, a chronic inflammatory skin disease in which the proliferation of keratinocytes presents as the main histopathologic feature and the secretion of chemokines from keratinocytes mediates the skin infiltration of T cells^17^, both consistent with our previous in vitro findings. To test the level of miR-17-92 cluster in psoriasis lesions, we collected skin specimens from 25 psoriasis patients and 25 healthy controls. The level of miR-17-92 cluster was not changed in perilesional skin tissues compared with control. However, lesional mir-17-92 level was significantly higher than that in healthy skin tissues or perilesional skin tissues (Fig. 5a). Moreover, lesional miR-17-92 level showed positive correlation with Psoriasis Area and Severity Index (PASI) score in the patients (Fig. 5b), indicating the involvement of miR-17-92 cluster in psoriasis development. To determine the source of increased miR-17-92 cluster in psoriasis lesions, we separated the epidermis and the dermis in lesional skin tissues from 10 patients, and examined miR-17-92 level in each part. We found that miR-17-92 cluster was mainly expressed in the epidermis layer rather than the dermis layer (Fig. 5c), implying that the increased mir-17-92 cluster in psoriatic lesions was from keratinocytes since they are the main cell content in the epidermis. Consistent with the level of miR-17-92 cluster, the levels of miR-17, miR-18a, miR-19a, miR-19b, miR-20a, and miR-92a were all upregulated in psoriasis lesions, compared with that in perilesional skin tissues or healthy skin tissues (Fig. 5d). Consequently, the expression of miR-17-92 cluster is increased in psoriasis lesions, mainly at the epidermis layer. We additionally examined the protein levels of CDKN2B and SOCS1, the two target genes responsible for the effects of miR-17-92 cluster on keratinocytes, in the skin specimens from five psoriasis patients and five healthy controls using immunofluorescence. The assay showed that the expression of CDKN2B was obviously repressed in psoriatic epidermis (Supplementary Figures S6A), which coincided with the increase of epidermal miR-17-92 cluster in psoriasis. Not surprisingly, the expression of SOCS1 showed elevation in psoriatic epidermis (Supplementary Figures S6B), probably because cytokines-induced SOCS1 upregulation could not be completely reversed by miR-17-92 elevation in psoriasis lesions.

### MiR-17-92 cluster can be upregulated by cytokines via the activation of STAT1 signaling pathway in keratinocytes

**Fig. 6:**
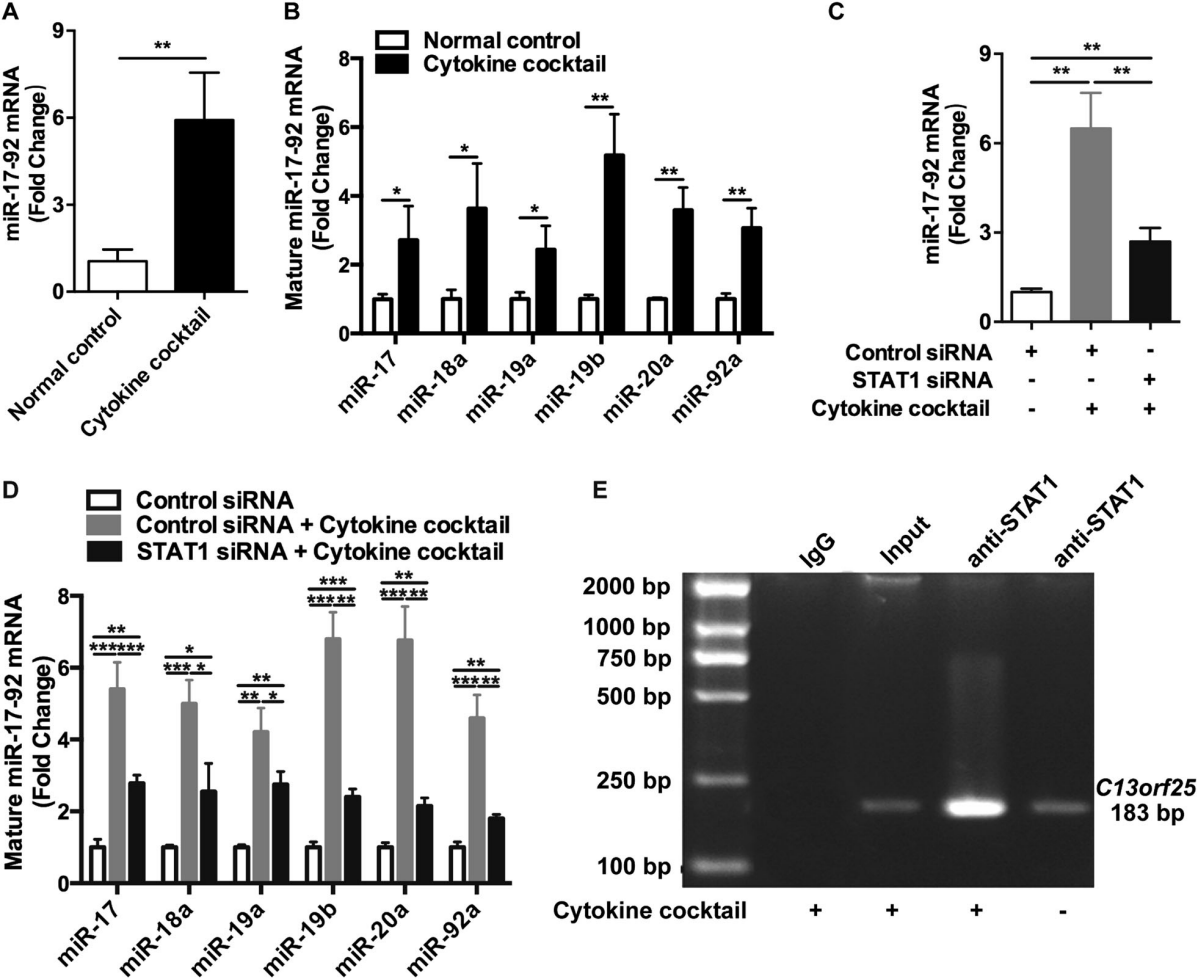
MiR-17-92 cluster could be upregulated by cytokines via the activation of STAT1 signaling pathway in keratinocytes. **a**, **c** The level of miR-17-92 cluster in NHKs with different transfections and treatments as indicated was analyzed using qRT-PCR. Mean ± SD is shown. Data are representative of three independently performed experiments. **P < 0.01. **b**, **d** The levels of miR-17, miR-18a, miR-19a, miR-19b, miR-20a, and miR-92a in NHKs with different transfections and treatments as indicated were analyzed using qRT-PCR. Mean ± SD is shown. Data are representative of three independently performed experiments. *P < 0.05; **P < 0.01; ***P < 0.001. **e** ChIP assay of NHKs treated by cytokine cocktail. Binding of STAT1 to the promoter region of *C13orf25* was confirmed by PCR with specific primers

We went on to investigate the potential mechanism underlying the increase of miR-17-92 cluster in psoriasis. Initially, we performed bioinformatics analysis on the sequence of *C13orf25* promoter region to predict potentially binding transcriptional factors using the JASPAR database (http://jaspar.genereg.net). As a result, STAT1, the transcriptional factors that had been demonstrated to be activated by cytokines in the keratinocytes of psoriatic lesion^40^, was predicted to bind to the promoter region of *C13orf25* (Supplementary Figures S7). We therefore stimulated NHKs with cytokine cocktail and tested the level of miR-17-92 cluster. The levels of miR-17-92 cluster and the six mature miRNAs derived from miR-17-92 cluster significantly increased in NHKs stimulated by cytokines (Fig. 6a, b). To testify the contribution of STAT1 to cytokines-induced miR-17-92 production, keratinocytes were first transfected with STAT1 siRNA or the negative control siRNA for 24 h (interference efficiency seen in Supplementary Figures S8) and then stimulated by cytokine cocktail. The following qRT-PCR assay showed significantly lower levels of miR-17-92 cluster and mature miRNAs in the cells transfected with STAT1 siRNA compared with control under the stimulation of cytokines (Fig. 6c, d). At last, our chromatin immunoprecipitation (ChIP) assay showed that more STAT1 proteins were recruited to *C13orf25* promoter region in the keratinocytes stimulated by cytokine cocktail (Fig. 6e). Taken together, our findings demonstrate that STAT1 signaling pathway is responsible for the increase of miR-17-92 cluster induced by cytokines in keratinocytes, which is possibly the mechanism underlying the upregulation of miR-17-92 cluster in psoriasis.

## Discussion

The benign proliferation of keratinocytes is a key event in the development of many chronic inflammatory cutaneous diseases, such as psoriasis, atopic dermatitis, and lichen planus^17–19^. Previous studies have stated that keratinocyte growth is closely related to the expression profile of keratins, with keratin 6, keratin 16, and keratin 17 highly expressed in hyperproliferative keratinocytes^41^. In chronic inflammatory skin diseases like psoriasis, the proliferation of keratinocytes can be induced by cytokines like IL-22 and interleukin-20^42^. Supplementary to these previous findings, our study demonstrates that miR-17-92, a miRNA cluster that can be increased by cytokines via STAT1 signaling pathway in keratinocytes, inhibits the expression of CDKN2B and removes cell-cycle arrest, which ultimately leads to the proliferation of keratinocytes. It is reported that miR-17-92 cluster promotes the cell-cycle progression of malignant cells in a number of cancer diseases, which results from the inhibition on a series of tumor suppressor genes including p21 and p53^43,44^. In addition to these reported target genes, our findings prove that CDKN2B is another tumor suppressor gene that could be suppressed by multiple mature miRNAs derived from miR-17-92 cluster. Therefore, the oncogenic role of miR-17-92 cluster in malignancy may result from, at least partially, its direct inhibition on CDKN2B, which is worth attention from oncologists in future studies.

The activation of keratinocytes is a crucial process in the development of chronic inflammatory skin diseases not only for the hyperproliferation but also for the immune function that further accelerates cutaneous inflammation. Activated keratinocytes can produce various inflammatory factors, and can even be the main sources of chemokines that directly lead to the cutaneous infiltration of immune cells in multiple diseases including psoriasis, vitiligo, and lichen planus^20,29,42^. In 2013, Xu et al. reported that miR-31 could promote the expressions of C-X-C motif chemokine ligand 1 (CXCL1), C-X-C motif chemokine ligand 5 (CXCL5), and C-X-C motif chemokine ligand 8 (CXCL8) in keratinocytes stimulated by TNF-α, all of which contribute to the migration of neutrophils^45^. Unlike their findings, our study demonstrates that miR-17-92 cluster is indispensible for cytokines-induced production of CXCL9 and CXCL10 in keratinocytes, both of which are important for the migration of effector T lymphocytes to skin. Therefore, miR-17-92 cluster is probably involved in the chemotaxis for T cells formed by keratinocytes in psoriasis.

The expression of miR-17-92 cluster is precisely regulated in malignant tumors by many transcriptional factors including MYC proto-oncogene, bHLH transcription factor (C-MYC), MYCN proto-oncogene, bHLH transcription factor (N-MYC), E2F transcription factor 1 (E2F1), and E2F transcription factor 3 (E2F3)^3^. As for the situation in autoimmune diseases, it is reported that miR-18a can be induced in synovial fibroblasts by TNF-α via nuclear factor of kappa light polypeptide gene enhancer in B-cells (NF-κB) signaling pathway in rheumatoid arthritis^32^, while the regulatory mechanism underlying in the expression of the whole miR-17-92 cluster in inflammatory conditions was not investigated before. Our findings prove that under the stimulation of cytokines, STAT1 can directly bind to the promoter region of *C13orf25* and transcriptionally facilitate the expression of miR-17-92 cluster in keratinocytes. Combined with our finding that miR-17-92 cluster inhibits the expression of SOCS1, a negative feedback regulator of STAT1 signaling pathway^37^, our study indicates a positive feedback loop between STAT1 activation and miR-17-92 upregulation, which may play a critical role in the pathological behavior of keratinocytes in inflammatory microenvironment.

Several miRNAs such as miR-21, miR-31, and miR-486-3p contribute to the development of psoriasis either through promoting the proliferation of keratinocytes or via enhancing cutaneous inflammation^25,46,47^. Our in vitro findings prove that miR-17-92 cluster can promote not only the proliferation but also the chemokine production of keratinocytes. Subsequent in vivo analysis did confirm that miR-17-92 cluster and all the six mature miRNAs derived from miR-17-92 cluster are increased in psoriatic lesions, especially in lesional epidermis, and the lesional level of miR-17-92 cluster is positively correlated with the disease severity of psoriasis. Taken together, our study indicates the involvement of miR-17-92 cluster in the pathogenesis of psoriasis. Recently, Wu et al. reported that specific deletion or overexpression of miR-17-92 cluster in keratinocytes failed to affect the development of imiquimod-induced psoriasis-like mice. According to their results, the expressions of miR-18a and miR-92a in the lesions of imiquimod-induced psoriasis-like dermatitis were not increased, and the expressions of miR-17 and miR-19b showed over 200 folds of change compared with control^48^, which, however, is not consistent with the expression of miR-17-92 cluster in human psoriatic lesions as we observed. Therefore, the imiquimod-induced psoriasis-like mouse model may not be appropriate to test the function of miR-17-92 cluster in the development of psoriasis. Further studies using other psoriasis mouse models such as the K5-STAT3 psoriasis-like mouse model and the IL-23-induced psoriasis-like mouse model^49^ are needed to fully clarify the role of miR-17-92 cluster in the pathogenesis of psoriasis.

In summary, we demonstrate the function of miR-17-92 cluster in promoting the proliferation and the chemokine production of keratinocytes. Moreover, miR-17-92 cluster is increased in psoriasis and can be elevated by cytokines in keratinocytes via STAT1 signaling pathway. Our study suggests that miR-17-92 cluster may contribute to the pathology of keratinocytes in chronic inflammatory skin diseases and thus play a role in the development of these diseases, especially psoriasis. Further in vivo studies are needed to evaluate the potential of miR-17-92 cluster as a therapeutic target for psoriasis or other skin diseases with similar inflammatory pathogenesis.

## Materials and Methods

### Human skin samples

Lesional and perilesional specimens were redundant tissues from 25 psoriasis patients who accepted biopsy. Among them, 10 larger lesional specimens were cut into halves, with one half of each specimen incubated with 2.5 mg/ml dispase (Gibco, Grand Island, NY, USA) for 8 h at 4 °C to separate the epidermis from the dermis before subsequent RNA isolation. The disease severity of the patients was evaluated using a PASI scoring system as previously described^50^. All the patients had no other autoimmune or systemic diseases, and none of the patients received systemic treatment, including glucocorticoids, immunomodulating agents, and phototherapy, within 1 month prior to the sample collection period. Healthy skin specimens were collected from 25 people who accepted cosmetic surgery at Xijing Hospital. All the specimens were partly paraffinized for further histological analysis. All of the patients and control subjects were pair-matched in age and gender, and informed consent was obtained from all the patients and control subjects. The research protocol was designed and executed according to the principles of the Declaration of Helsinki and was approved by the ethics review board of Fourth Military Medical University.

### Cell culture and cytokine stimulation

NHKs were extracted from prepuces obtained from healthy individuals who accepted circumcision. Informed consent was obtained from all donors. Keratinocytes were cultured in the serum-free keratinocyte growth medium (Gibco), and the second- or third-passage keratinocytes were used in all experiments. Cytokine cocktail used for stimulating NHKs was composed of recombinant human TNF-α (50 ng/ml), recombinant human IFN-γ (10 ng/ml), recombinant human IL-17 (25 ng/ml), and recombinant human IL-22 (25 ng/ml) (Peprotech, Rocky Hill City, CT, USA). Peripheral blood mononuclear cells (PBMCs) were obtained from psoriasis patients with informed consent and cultured in Modified Medium RPMI 1640 (HyClone, Logan, UT, USA) with 10% fetal bovine serum (Sijiqing, Hangzhou, China) before subsequent transwell assay. Each experiment was repeatedly performed using the cells at least from three different sources.

### Plasmid transfection and RNA interference

The Plamid used for the overexpression of miR-17-92 cluster was pcDNA3.1-miR-17-92 plasmid (Addgene, Cambridge, MA, USA), with pcDNA3.1 empty plasmid (Genechem, Shanghai, China) as the control. The plasmids used for rescue experiments included pCMV6-CDKN2B plasmid, pCMV6-SOCS1 plasmid, and pCMV6 empty plasmid (Origene, Rockville, MD, USA). The siRNAs used for STAT1 knockdown were STAT1 p84/p91 siRNA and Normal Control siRNA (Santa Cruz, CA, USA). The siRNAs used for miR-17-92 knockdown were miR-17-92 siRNA and Negative Control siRNA (RiboBio, Guangzhou, China). All the experiments with plasmid transfection or RNA interference were performed using Lipofectamine 3000 (Invitrogen, Carlsbad, NM, USA) following the manufacturer’s protocol.

### RNA isolation and qRT-PCR analysis

Total RNA from skin specimens or culture cells was isolated using Trizol reagent (Invitrogen), and then reversely transcribed to cDNA by using PrimeScript RT reagent Kit (Takara, Ohtsu, Japan) for mRNA or by using miRNA cDNA First Strand Synthesis (Tiangen, Beijing, China) for miRNA. QRT-PCR for cDNA samples reversely transcribed from mRNA was performed using SYBR Premix Ex Taq II (TaKaRa). The relative mRNA expression was normalized to the actin gene. The primer sequences are listed in Supplementary Tables S1. QRT-PCR for cDNA samples reversely transcribed from miRNA was performed using miRcute miRNA Fluorescence Quantitation Kit (Tiangen). The relative miRNA expression was normalized to RUNU6. The primers of miR-17, miR-18a, miR-19a, miR-19b, miR-20a, miR-92a, and RUNU6 were designed and synthesized by Tiangen company based on based on miRBase (http://www.mirbase.org), with the primer sequences as commercially confidential data. All the qRT-PCR assays were performed with the iQ5 PCR Detection System (Bio-Rad, Berkeley, CA, USA). Threshold cycle (CT) values were used to calculate the fold change by using the 2^−ΔΔCT^ method.

### Cell viability Assay

Cell viability was determined by the Cell Counting Kit-8 (CCK8) assay (7seabiotech, Shanghai, China). According to the manufacturer’s instructions, 4 × 10^3^ cells were seeded in each well of a 96-well culture plate overnight. After synchronization, cells were prepared for indicated procedures. At 12 h, 24 h and 48 h, the cells were incubated with CCK8 reagents for 1 h. The microplates were then read in a plate reader (Bio-Rad) at a wavelength of 450 nm. Each sample was analyzed in triplicate.

### 5-ethynyl-2′-deoxyuridine assay

For 5-ethynyl-2′-deoxyuridine (EdU) analysis, 2 × 10^3^ cells were seeded in each well of a 96-well culture plate overnight. After synchronization, the cells were prepared for indicated procedures. At 24 h, the cells were harvested and Edu assay was performed using Cell Light EdU DNA imaging Kit (RiboBio) according to the manufacturer’s instructions. Images were taken and analyzed using High Content Imaging Pathway 855 (BD, Franklin Lakes, NJ, USA). EdU-positive cells were calculated by (EdU add-in cells/Hoechst stained cells) × 100%.

### Detection of apoptosis

To quantify apoptotic death, the cells were seeded in six-well plates at a density of 2 × 10^5^ cells per well overnight. After indicated transfections, the cells were collected and the apoptotic portion was identified using the Annexin V-PE Apoptosis Detection Kit (7seabiotech) following the manufacturer’s instructions. The number of apoptotic cells was then quantified by flow cytometry (Beckman Coulter, Brea, CA, USA), and the analysis was carried out using the Expo32 software (Beckman Coulter).

### Cell-cycle analysis

Cells were seeded in 6-well culture plates overnight. After synchronization, cells were prepared for following procedures. 24 h later, the cells were harvested from each well by trypsinization and fixed in 70% (v/v) cold ethanol at 4 °C overnight. After washing with ice-cold PBS, the fixed-cell pellets were collected by centrifugation and resuspended in PI/RNase Staining Buffer (7seabiotech) for staining of DNA and finally analyzed using a flow cytometer (Beckman Coulter).

### Western blotting

Cells were lysed with cell lysis solution (DSL, Webster, USA). The total protein concentration in lysates was measured with BCA Protein Assay kit (Pierce, Rockford, IL, USA). Equal amounts of protein were separated by 10% SDS-PAGE (Bio-Rad) and were then transferred from the gel to polyvinylidene difluoride membranes (Millipore, Billerica, MA, USA). Page Ruler Plus Prestained Protein Ladder (Fermentas, Hanover, MD, USA) was used to confirm protein electrophoresis and transferring. After blocking in a solution of 5% non-fat dry milk diluted in tris-buffered saline, the membranes were washed and then incubated with primary antibodies (Supplemental Tables S2) overnight at 4 °C. After being washed, the membranes were incubated with horseradish peroxidase-conjugated secondary antibodies (Goat Anti-Mouse IgG, 1:5000; Goat Anti-Rabbit IgG, 1:10000) for 2 h at room temperature. Bound antibodies were detected using the ECL western blotting detection system (Millipore).

### Luciferase reporter assay

Cells were seeded in 6-well culture plates overnight. After synchronization, luciferase vectors (Genechem) were individually transfected into the cells along with miR-17-92 plasmid or pcDNA3.1 plasmid using Lipofectamine 3000 according to the manufacturer’s recommendations. After 48 h of transfection, luciferase assays were performed using the Dual-Luciferase Reporter Assay System (Promega, Madison, WI, USA). For each transfection, the luciferase activity of three replicates was averaged.

### ELISA assay

ELISA analysis on the culture medium of NHKs with indicated treatments or transfections for 48 h was performed using Human CXCL9 ELISA Kit (R&D system, Minneapolis, MN, USA) and Human CXCL10 ELISA Kit (R&D system) according to the manufacturer’s instructions, respectively. The absorbance (A450) was measured with a plate reader (Bio-Rad).

### Transwell assay

NHKs were treated or transfected as indicated. After 48 h, culture supernatants were collected for the following chemotaxis assay. PBMCs–derived psoriasis CD3^+^ T cells (1 × 10^5^ cells, cultured in 100 μL medium) were sorted for the chemotaxis assay, separated from the cell supernatants by 5.0 μm polycarbonate membrane (Corning Life Sciences, Corning, NY, USA). In addition, CXCL9 neutralization antibody (R&D Systems) and CXCL10 neutralization antibody (R&D Systems) were used to bloke CXCL9 or CXCL10. Plates were left at 37 °C for 3 h. Migrated cells in the lower chamber were counted by flow cytometry (Beckman Coulter).

### Immunofluorescence

For skin specimens, deparaffinized 5-μm tissue sections were first through heat-mediated antigen retrieval with Tris-EDTA buffer (PH 9.0). Subsequently, the skin sections were blocked at room temperature with 5% goat serum in PBS for 30 minutes, and incubated with primary antibodies (Supplemental Table S2) at 4 °C overnight, followed by 1 h incubation with secondary Goat anti-Rabbit IgG, Red Cy3 Conjugated, 1:200 (Cwbio, Beijing, China) at room temperature. Dapi (Dako, Glostrup, Denmark) was used to mark cell nucleus. All the specimens were analyzed by FV-1000/ES confocal microscope (Olympus America, Melville, NY, USA).

### Chromatin immunoprecipitation assay

ChIP assay was performed using a ChIP assay kit (Millipore) according to the manufacturer’s instructions. Briefly, the cells were incubated in 1% formaldehyde for 10 min at room temperature to crosslink their DNA. The cells were lysed in lysis buffer, sonicated to generate DNA fragments less than 500 base pairs in length and then diluted by ten folds in ChIP Dilution Buffer. Before immunoprecipitation, nuclear extracts were pre-cleared with 50% protein G-Sepharose slurry, goat normal serum, and sheared salmon sperm DNA for 2 h at 4 °C. Anti-STAT1 antibody (Cell Signaling Technology, Beverly, MA, USA) was then added to form complexes with STAT1 protein and associated chromatin. These immunocomplexes were recovered by protein G-Sepharose beads, and the associated DNA was purified by extraction with phenol/chloroform. Specific primers forward 5′- ACGGATTTGCTAATTTTAAGGTAGT -3′ and reverse 5′- TCAGTCAATTGTAGCAGAACGTA -3′ were used to measure the enrichment of the putative STAT1-binding site in the *C13orf25* promoter.

### Statistical analysis

Each experiment was performed at least for three times, and statistical analyses of the data were mainly performed using unpaired two-tailed Student’s *t*-test built into GraphPad Prism (GraphPad Software 3.0; San Diego, CA, USA). Notably, the statistical analysis on the expression of miR-17-92 between lesional epidermis and lesional dermis was performed using paired two-tailed Student’s *t*-test, and correlation analysis was performed by Spearman’s rank correlation test. *P*-values of <0.05 were considered statistically significant.

## Electronic supplementary material

Supplementary Figure Legends

Supplementary Tables S1

Supplementary Tables S2

Supplementary Figures S1

Supplementary Figures S2

Supplementary Figures S3

Supplementary Figures S4

Supplementary Figures S5

Supplementary Figures S6

Supplementary Figures S7

Supplementary Figures S8
