# Supplementary material for: MicroRNA-17-92 cluster promotes the proliferation and the chemokine production of keratinocytes: implication for the pathogenesis of psoriasis
Source: Cell Death Dis. 2018 May 11;9(5):567. doi: 10.1038/s41419-018-0621-y (PMC5948221; doi:10.1038/s41419-018-0621-y)
Supplement: Supplementary file 1 — Supplementary Figure Legends [file 41419_2018_621_MOESM1_ESM.doc]

**Supplementary Figures Legends**

**Supplementary Figures S1.** Transfection efficiency of miR-17-92 plasmid. The levels of (**A**) miR-17-92 cluster as well as (**B**) the six mature miRNAs including miR-17, miR-18a, miR-19a, miR-19b, miR-20a and miR-92a in NHKs transfected with miR-17-92 plasmid or the control pcDNA3.1 plasmid were analyzed using qRT-PCR. Values represent mean ± SD of three independent experiments. **P* < 0.05; ***P* < 0.01; ****P* < 0.001.

**Supplementary Figures S2.** MiR-17-92 cluster has no influence on the apoptosis of keratinocytes. (**A**) Flow cytometry analysis of apoptosis by Annexin V (an indicator of apoptosis) and PI staining in NHKs transfected with miR-17-92 plasmid or the control pcDNA3.1 plasmid. (**B**) The statistical chart represents 3 individual experiments. Data are presented as means ± SD. ns, not significant.

**Supplementary Figures S3.** The predicted target sites in the 3’UTR of CDKN2B mRNAs can be bound by miR-17-92 cluster-derived mature miRNAs. (**A**) The sequences of miR-17, miR-18a, miR-19a and miR-19b and their complementary sequences in 3’UTR of CDKN2B mRNA. (**B**-**D**) The construction strategy for the luciferase reporter plasmids of CDKN2B. Red letters represent the nucleotides that we mutated. The structures of the luciferase reporter plasmids are shown below. (**E**) NHKs were co-transfected by CDKN2B WT (No.1, No.2, No.3, respectively), CDKN2B MUT (No.1, No.2, No.3, respectively) or the control vector with miR-17-92 plasmid or the control pcDNA3.1 plasmid for 48 hours. Fireﬂy luciferase levels were then detected. Data are expressed as the ratio of luciferase activity detected in cells transfected by miR-17-92 plasmid to that in cells transfected by the control pcDNA3.1 plasmid. **P* < 0.05; ***P* < 0.01.

**Supplementary Figures S4.** The knockdown of MiR-17-92 cluster blocks the production of chemokines in keratinocytes primed by cytokines. The levels of (**A**) miR-17-92 cluster as well as (**B**) the six mature miRNAs including miR-17, miR-18a, miR-19a, miR-19b, miR-20a and miR-92a in NHKs transfected with miR-17-92 siRNA or the control siRNA were analyzed using qRT-PCR. Values represent mean ± SD of three independent experiments. ***P* < 0.01; ****P* < 0.001. (**C**) The mRNA levels of CXCL9 and CXCL10 in NHKs with different treatments and transfections as indicated were analyzed using qRT-PCR. Mean ± SD is shown. Data are representative of three independently performed experiments. **P* < 0.05; ***P* < 0.01; ****P* < 0.001. (**D**) The culture mediums of NHKs with different treatments and transfections as indicated were analyzed by ELISA to determine the secretion level of CXCL9 and CXCL10. Mean ± SD is shown. Data are representative of three independently performed experiments. ***P* < 0.01. (**E**) The migrations of CD3^+^ T cells in response to the culture mediums from NHKs with different treatments and transfections as indicated were evaluated using transwell assay. Mean ± SD is shown. Data are representative of three individual experiments. **P* < 0.05; ***P* < 0.01.

**Supplementary Figures S5.** The predicted target sites in the 3’UTR of SOCS1 mRNAs can be bound by miR-17-92 cluster-derived mature miRNAs. (**A**) The sequences of miR-19a and miR-19b and their complementary sequences in 3’UTR of SOCS1 mRNA. (**B**) The construction strategy for the luciferase reporter plasmids of SOCS1. Red letters represent the nucleotides that we mutated. The structures of the luciferase reporter plasmids are shown below. (**C**) NHKs were co-transfected by SOCS1 WT, SOCS1 MUT or the control vector with miR-17-92 plasmid or the control pcDNA3.1 plasmid for 48 hours. Fireﬂy luciferase levels were then detected. Data are expressed as the ratio of luciferase activity detected in cells transfected by miR-17-92 plasmid to that in cells transfected by control pcDNA3.1 plasmid. ***P* < 0.01.

**Supplementary Figures S6.** The protein levels of CDKN2B and SOCS1 are altered in psoriatic epidermis. The expressions of (**A**) CDKN2B and (**B**) SOCS1 were shown in the specimens of lesional skin (LS) and peri-lesional skin (PLS) from psoriasis patients as well as healthy skin (HS) from healthy donors using immunofluorescence. The second antibody was marked with Cy3 (red). Nuclei were counterstained with DAPI (blue). Scale bar = 50 um.

**Supplementary Figures S7.** Bioinformatics analysis on the binding site of STAT1 in the promoter region of *C13orf25* gene. The online computational tool JASPAR was used to perform the bioinformatics analysis. Human *C13orf25* promoter fragment is shown. The red bar represents the specific site that may be bound by STAT1.

**Supplementary Figures S8.** Interference efficiency of STAT1 siRNA. The levels of STAT1 and p-STAT1 in NHKs transfected with STAT1 siRNA or the control siRNA were detected by western blotting. β-actin was detected as loading control. Data are representative of three independently performed experiments.
