# Supplementary material for: MicroRNA-17-92 cluster promotes the proliferation and the chemokine production of keratinocytes: implication for the pathogenesis of psoriasis
Source: Cell Death Dis. 2018 May 11;9(5):567. doi: 10.1038/s41419-018-0621-y (PMC5948221; doi:10.1038/s41419-018-0621-y)
Supplement: Supplementary file 2 — Supplementary Tables S1 [file 41419_2018_621_MOESM2_ESM.docx]

**Supplementary Tables S1. Sequence information for real time PCR primers used in described studies.**

| **Gene** | **Primers (5′→3′)** |
| --- | --- |
| miR-17-92 | F^1^: CAGTAAAGGTAAGGAGAGCTCAATCTG  R^2^: CATACAACCACTAAGCTAAAGAATAATCTGA |
| CCL20 | F: TACTCCACCTCTGCGGCGAATCAGAA  R: GTGAAACCTCCAACCCCAGCAAGGTT |
| CCL27 | F: CTCTACCGAAAGCCACTCTCA  R: GAAGCACGAAAGCCTGGA |
| CXCL9 | F: GAGGGCAAGAGCCACAGTAT  R: TGGAGTAGCCAGGAAAGAGC |
| CXCL10 | F: CCTCCAGTCTCAGCACCAT  R: AAATTGGCTTGCAGGAATA |
| CXCL11 | F: TGAGTGTGAAGGGCATGGCT  R: GCTTTTACCCCAGGGCCTAT |
| CX3CL1 | F: GAGCCGACTCCTTCTTCCC  R: CCCTCCATCCTGAGCCTTT |
| Actin | F: AGAAAATCTGGCACCACACC  R: AGAGGCGTACAGGGATAGCA |

^1^F (forward primer), ^2^R (reverse primer)
