# Supplementary material for: MicroRNA-17-92 cluster promotes the proliferation and the chemokine production of keratinocytes: implication for the pathogenesis of psoriasis
Source: Cell Death Dis. 2018 May 11;9(5):567. doi: 10.1038/s41419-018-0621-y (PMC5948221; doi:10.1038/s41419-018-0621-y)
Supplement: Supplementary file 3 — Supplementary Tables S2 [file 41419_2018_621_MOESM3_ESM.docx]

**Supplementary Tables S2. Information for primary antibodies used in described studies.**

| **Antibodies** | **Company** | **Application** |
| --- | --- | --- |
| Rabbit anti-human CDKN2B antibody | Santa Cruz | WB^1^: 1:200 |
|  |  | IF^2^: 1:50 |
| Mouse anti-human Rb antibody | Cell Signaling Technology | WB: 1:1000 |
| Rabbit anti-human p-Rb antibody | Cell Signaling Technology | WB: 1:1000 |
| Rabbit anti-human SOCS1 antibody | Proteintech | WB: 1:1000 |
|  |  | IF: 1:100 |
| Rabbit anti-human STAT1 antibody | Cell Signaling Technology | WB: 1:1000 |
| Rabbit anti-human p-STAT1 antibody | Cell Signaling Technology | WB: 1:1000 |
| Rabbit anti-human IRF-1 antibody | Cell Signaling Technology | WB: 1:1000 |
| Mouse anti-human β-actin antibody | Cwbio | WB: 1:5000 |

^1^WB (western blot), ^2^IF (immunofluorescence)
