# Supplementary figures and images for: MicroRNA-17-92 cluster promotes the proliferation and the chemokine production of keratinocytes: implication for the pathogenesis of psoriasis
Source: Cell Death Dis. 2018 May 11;9(5):567. doi: 10.1038/s41419-018-0621-y (PMC5948221; doi:10.1038/s41419-018-0621-y)

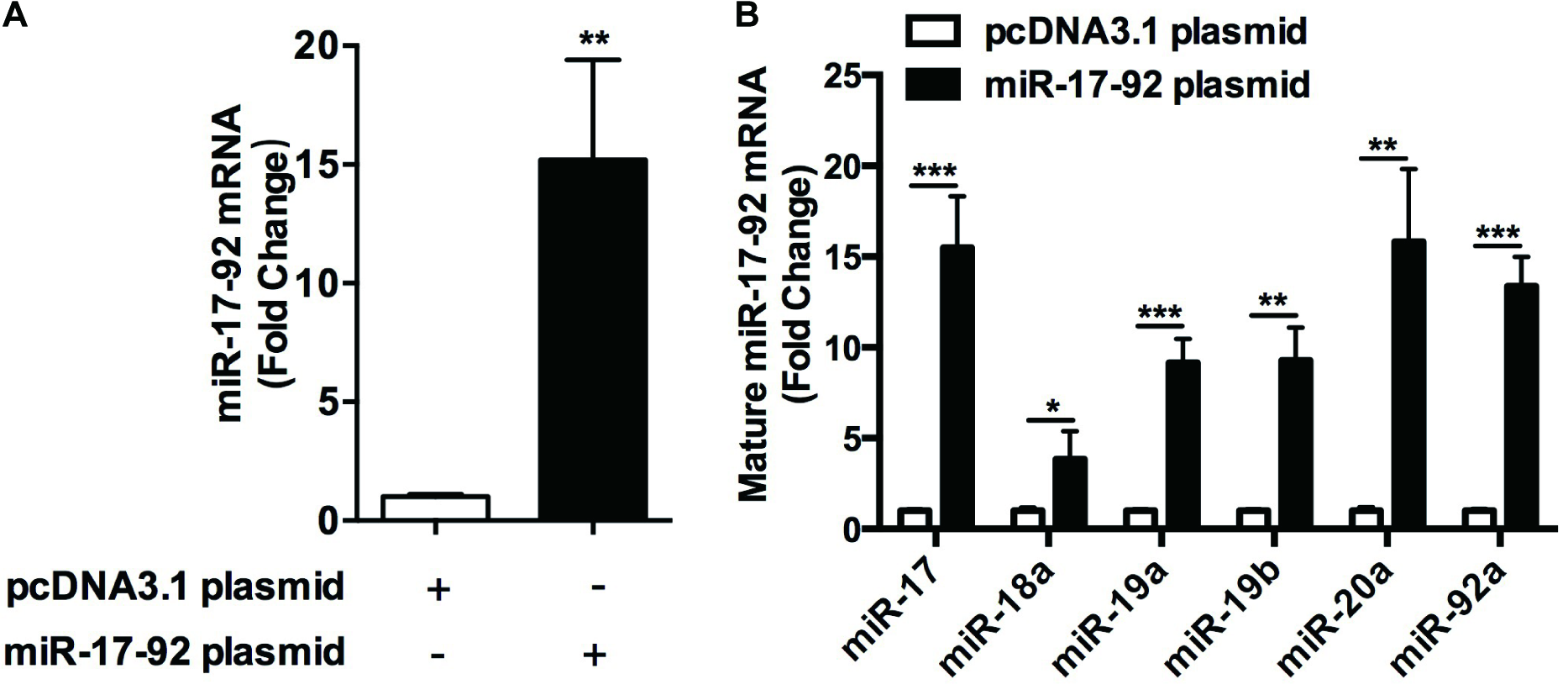

Supplement: Supplementary file 4 — Supplementary Figures S1 [file 41419_2018_621_MOESM4_ESM.tif]

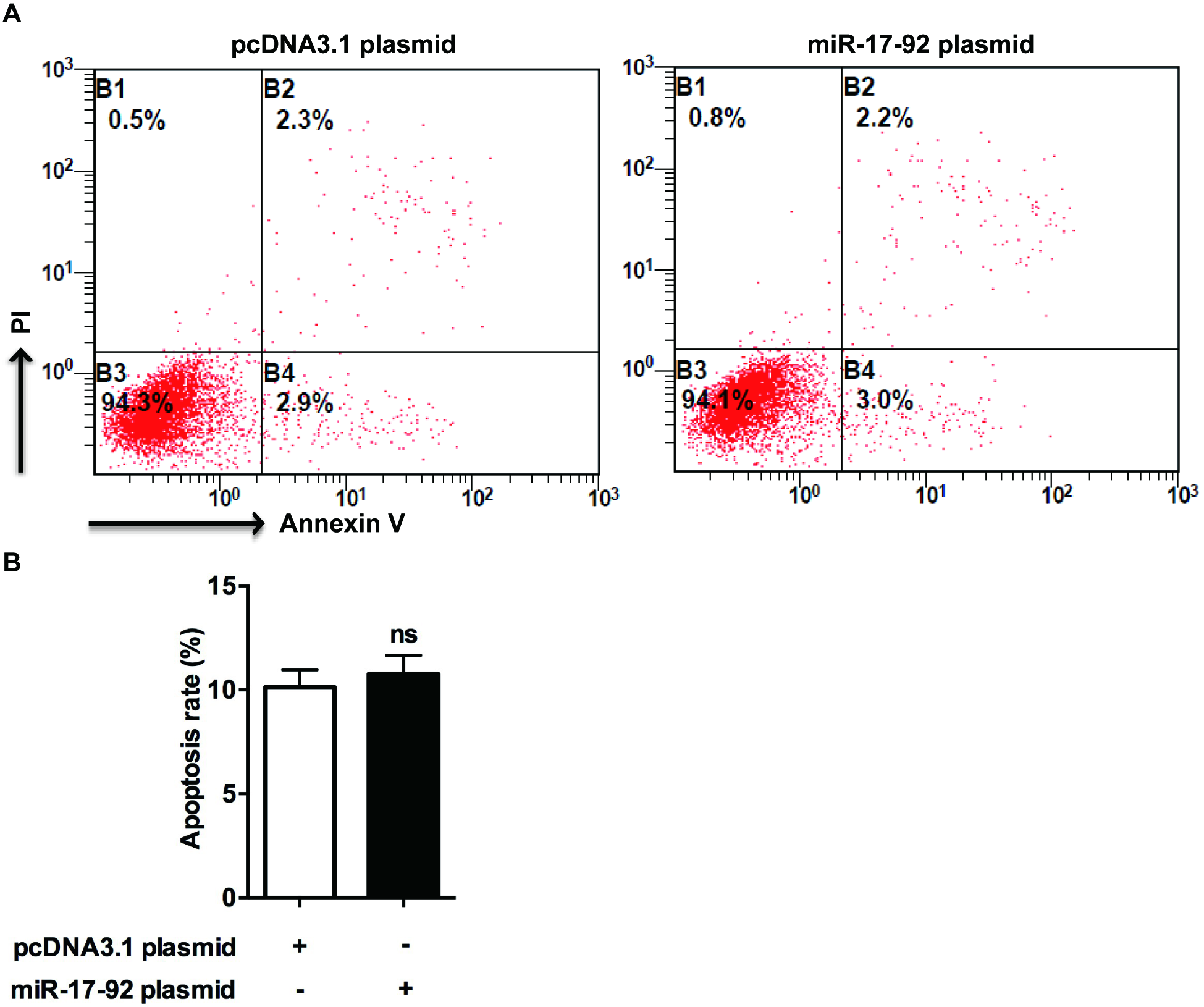

Supplement: Supplementary file 5 — Supplementary Figures S2 [file 41419_2018_621_MOESM5_ESM.tif]

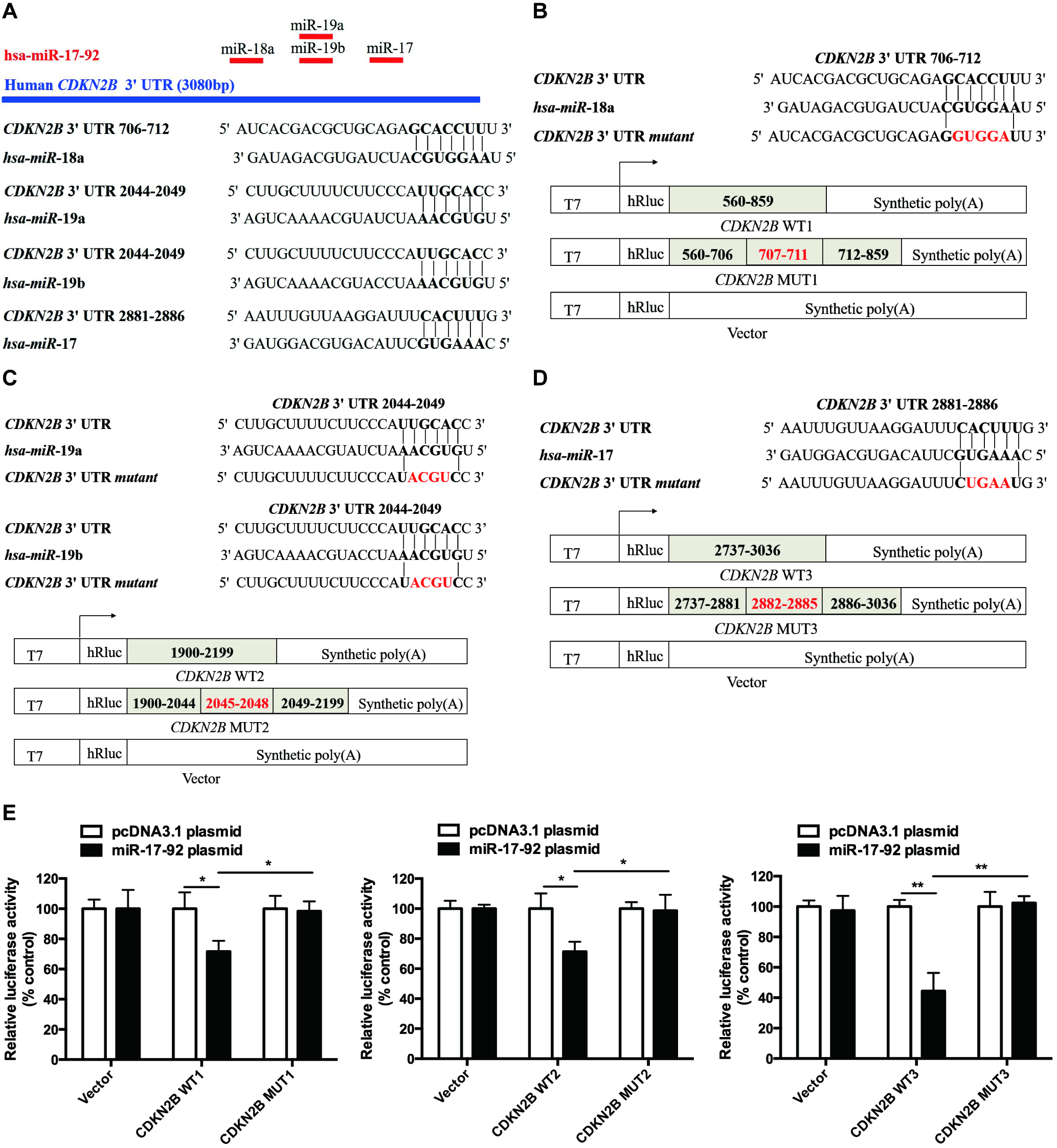

Supplement: Supplementary file 6 — Supplementary Figures S3 [file 41419_2018_621_MOESM6_ESM.tif]

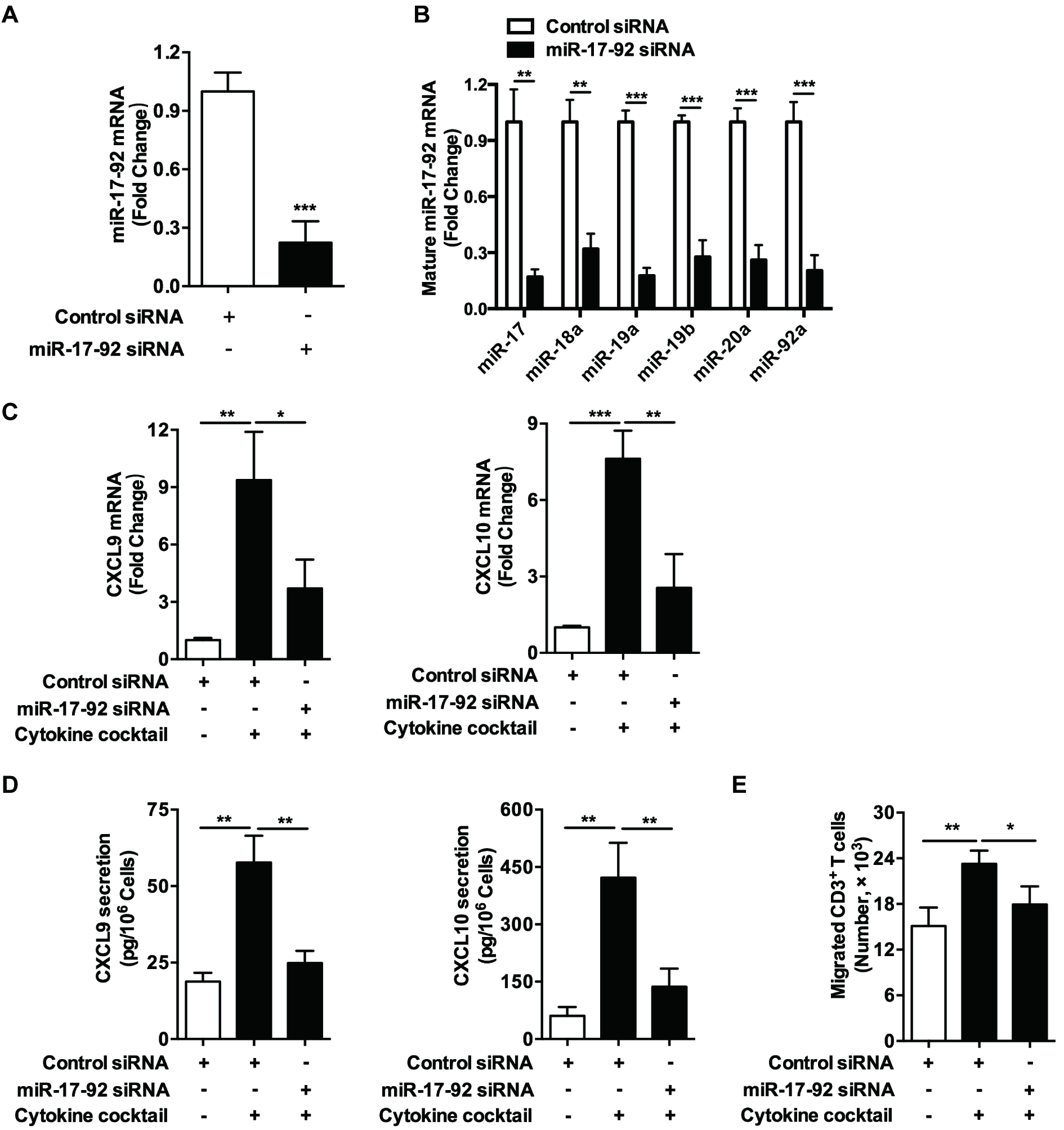

Supplement: Supplementary file 7 — Supplementary Figures S4 [file 41419_2018_621_MOESM7_ESM.tif]

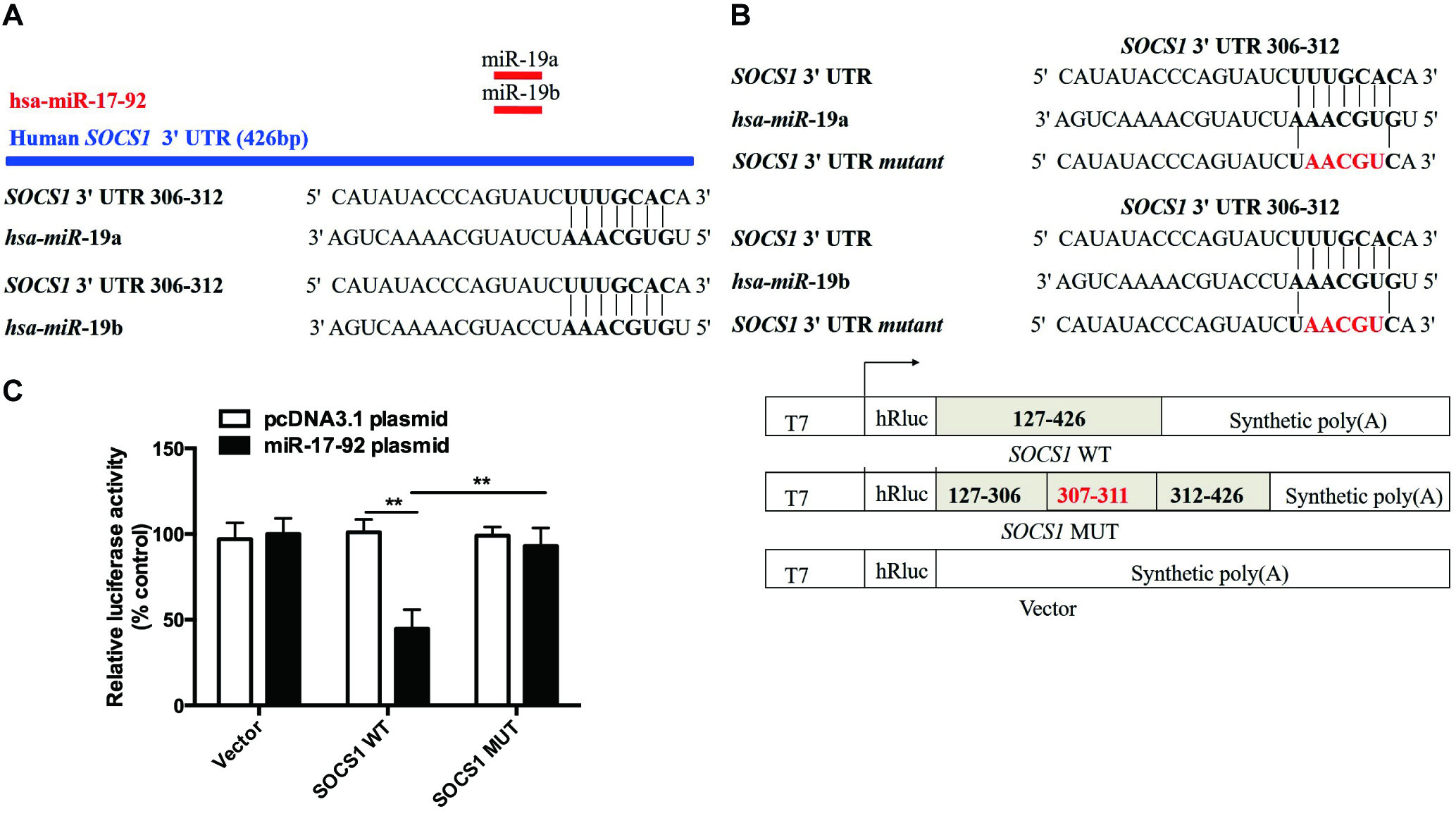

Supplement: Supplementary file 8 — Supplementary Figures S5 [file 41419_2018_621_MOESM8_ESM.tif]

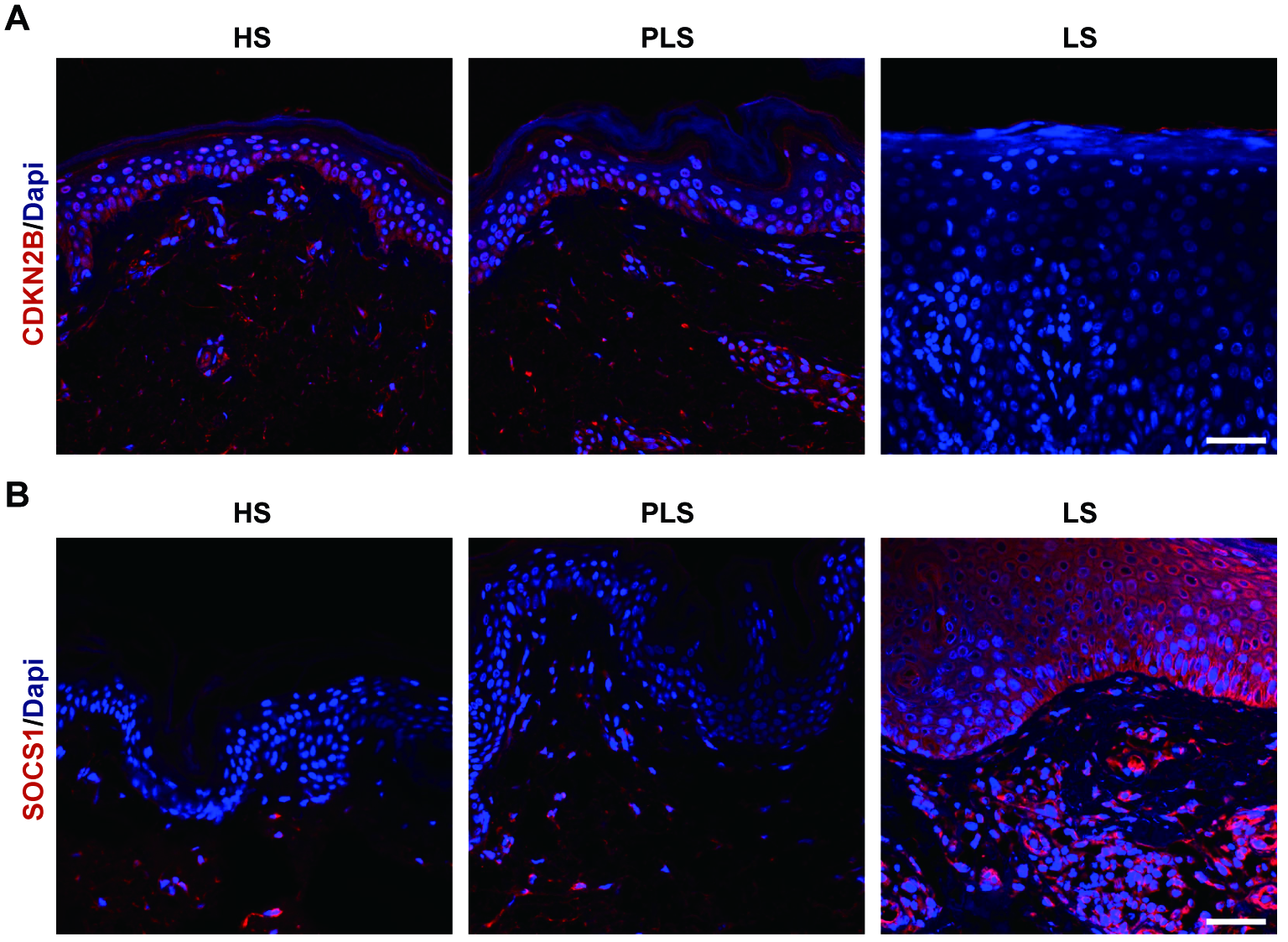

Supplement: Supplementary file 9 — Supplementary Figures S6 [file 41419_2018_621_MOESM9_ESM.tif]

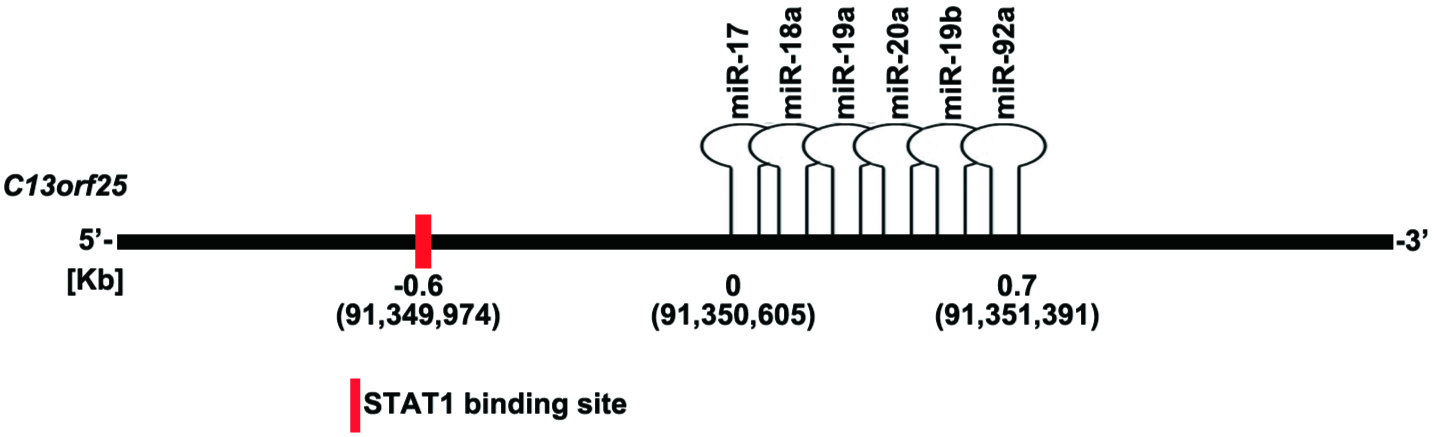

Supplement: Supplementary file 10 — Supplementary Figures S7 [file 41419_2018_621_MOESM10_ESM.tif]

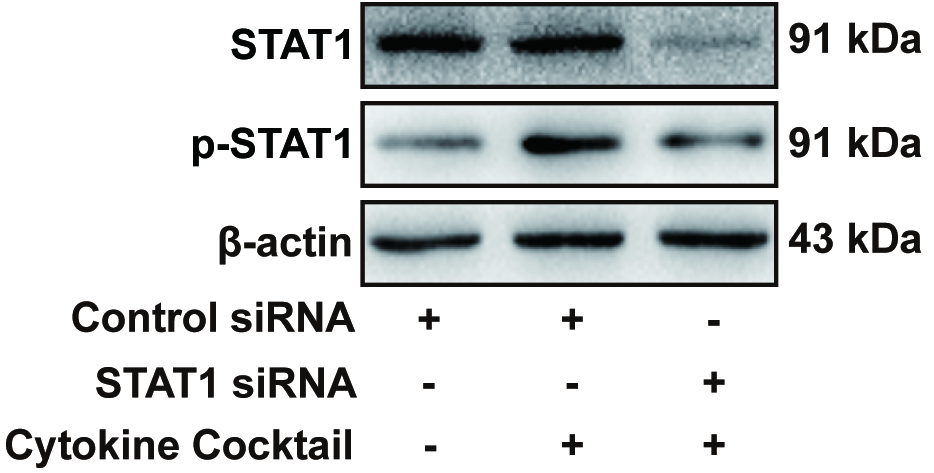

Supplement: Supplementary file 11 — Supplementary Figures S8 [file 41419_2018_621_MOESM11_ESM.tif]
